# Disseminated Nocardia paucivorans Infection Resembling Metastatic Disease in a Kidney Transplant Recipient

**DOI:** 10.7759/cureus.25365

**Published:** 2022-05-26

**Authors:** Tyler Luu, Rabeeya Khalid, Tyler Rehman, Nina M Clark

**Affiliations:** 1 Department of Medicine, Loyola University Chicago Stritch School of Medicine, Maywood, USA; 2 Division of infectious Diseases, Department of Medicine, Loyola University Medical Center, Maywood, USA; 3 Division of Infectious Diseases, Department of Medicine, Loyola University Chicago Stritch School of Medicine, Maywood, USA

**Keywords:** immunocompromised, kidney transplant, metastatic disease, disseminated nocardiosis, nocardia paucivorans

## Abstract

Recipients of solid-organ transplants (SOT) or hematopoietic stem-cell transplants are prone to various complications, including serious infections. Nocardiosis is an opportunistic bacterial infection that primarily affects the lung. It may also cause skin and soft-tissue infection, cerebral abscess, bloodstream infection, or infection involving other organs. We present a case of an immunocompromised kidney transplant recipient who experienced a prolonged history of unexplained indolent constitutional symptoms without a fever. Initial radiographic findings were suggestive of metastatic disease at multiple sites. However, metagenomic next-generation sequencing of microbial cell-free DNA in blood revealed disseminated *Nocardia paucivorans *infection, and organisms consistent with *Nocardia* were identified on histopathology of a lung biopsy. It is crucial for healthcare providers to be aware of unusual opportunistic infections to provide appropriate workups and interventions for immunocompromised SOT recipients.

## Introduction

Infections are a leading cause of morbidity and mortality among immunocompromised individuals [1]. Depending on the nature of their immunosuppression, patients may be at risk for various infectious complications, both common and rare [1]. Nocardiosis, an uncommon opportunistic infection, has been described in solid-organ transplant (SOT) recipients early on in transplantation [2], with kidney and liver transplant recipients at lower risk of acquiring nocardiosis compared to other SOT recipients such as lung and heart recipients [3]. Following pulmonary or cutaneous inoculation, *Nocardia* can disseminate to virtually any organ [4]. Disseminated nocardiosis is defined as the involvement of two noncontiguous anatomic sites that may or may not include a pulmonary focus [4]. The consequences of these infections may be devastating without prompt diagnosis and intervention [5]. Disseminated nocardiosis resembling metastatic disease in lung and kidney transplant recipients has rarely been reported in the literature [3,6]. In this article, we present a case of disseminated *Nocardia paucivorans* infection that resembled metastatic disease in an immunocompromised kidney transplant recipient.

## Case presentation

A 70-year-old man with a history of end-stage renal disease due to polycystic kidney disease had undergone deceased donor kidney transplant 13 years prior. He also had chronic thrombocytopenia with an unrevealing bone marrow biopsy a year prior, atrial fibrillation, and severe mitral valve regurgitation.

He presented to a tertiary medical center for a planned transcatheter mitral valve repair procedure; however, pre-procedural workup demonstrated acute kidney injury and pyuria, and blood and urine cultures grew *Klebsiella pneumoniae*. A comprehensive interview revealed a 150-pound unintentional weight loss over two years associated with intermittent rigors, night sweats, and dry cough. The patient’s immunosuppressive therapy at the time of admission included stable doses of tacrolimus and mycophenolate mofetil. He had been treated with plasmapheresis (PLEX) and high-dose prednisone for biopsy-proven antibody-mediated rejection one year prior to admission, and again received PLEX and high-dose steroids eight months prior to admission for hemolytic uremic syndrome in the setting of *Escherichia coli* sepsis.

**Figure 1:**
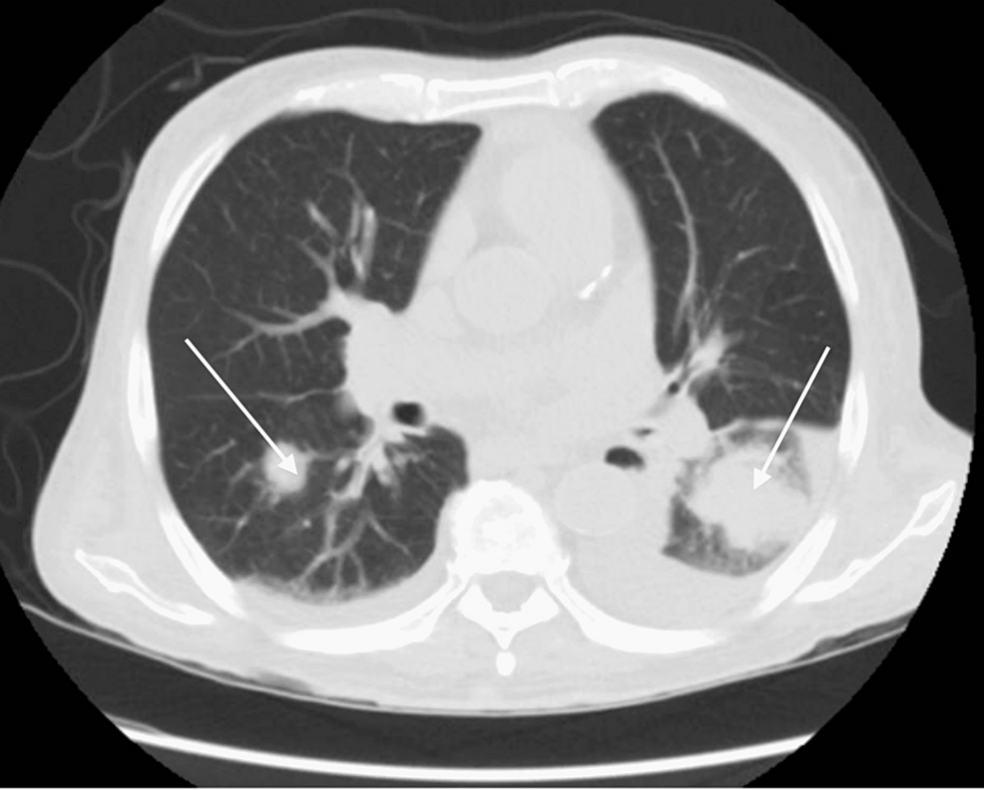
White arrows show left lower lobe infiltrate and right lung nodule on chest CT. CT: computed tomography

**Figure 2:**
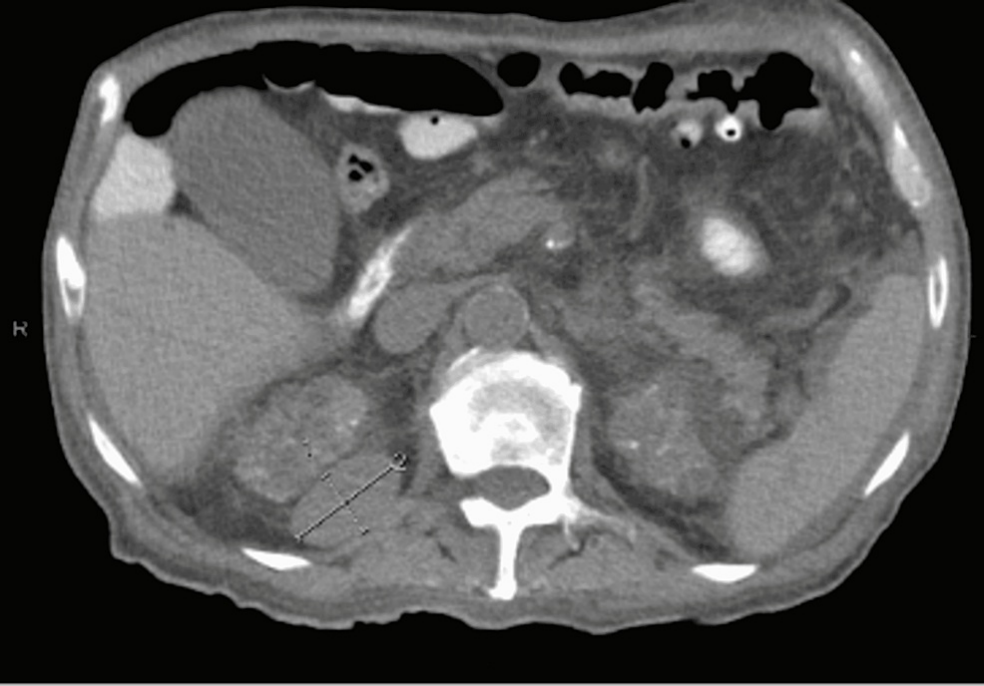
CT of the abdomen demonstrating a 4.1 cm × 2.5 cm left retroperitoneal nodule, suspicious for a metastatic lesion. CT: computed tomography

**Figure 3:**
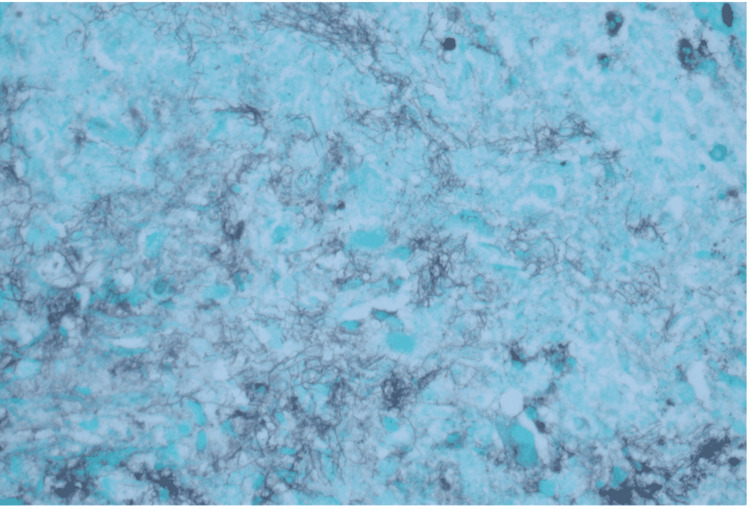
Grocott’s methenamine silver stain of lung biopsy at 10× magnification showing clumps of filamentous organisms, consistent with Nocardia.

Further investigation of his constitutional symptoms consisted of computed tomography (CT) scans of the chest, abdomen, and pelvis, which demonstrated a left lower lung infiltrate, multiple bilateral lung nodules measuring up to 4 cm in diameter, pleural effusions, and a 4.1 cm × 2.5 cm left retroperitoneal nodule, suspicious for metastatic disease (Figures 1, 2). The official radiographic interpretation was that the findings were suggestive of metastatic disease. Fine needle aspiration of the right retroperitoneal mass was performed but cytology of the aspirated fluid was of suboptimal cellularity; it showed rare inflammatory cells and debris but no malignancy. C-reactive protein was markedly elevated to 146 mg/L (normal range: less than 10 mg/L). The aerobic culture of the fluid was negative, but neither acid-fast bacillus (AFB) nor fungal culture was ordered on the aspirate. A transcatheter core biopsy of the left lung was then performed. Histology of the lung biopsy demonstrated foci of necrosis with inflammatory debris, collections of histiocytes, as well as acute and chronic inflammatory cells within the lung parenchyma. Grocott’s methenamine silver stain was initially read as negative but further review with the pathologist showed clumps of filamentous organisms suggestive of *Nocardia* (Figure 3) that had first been thought to represent debris. A lung biopsy culture was not ordered due to the strong suspicion of malignancy. Other radiographic workup included brain magnetic resonance imaging (MRI) which showed diffuse dural enhancement overlying both cerebral hemispheres, consistent with an infectious process. Lumbar puncture showed an elevated cerebrospinal fluid (CSF) protein level but no white blood cells. CSF meningitis polymerase chain reaction (PCR) panel and aerobic culture were negative as were blood AFB and fungal cultures and sputum fungal cultures. However, metagenomic next-generation sequencing (mNGS) of microbial cell-free DNA [7] was obtained and demonstrated *Nocardia paucivorans* at 4,007 DNA molecules/µL.

Based on the mNGS results, the histopathology findings, and radiographic evidence, the patient was diagnosed with disseminated *Nocardia paucivorans* infection. Antimicrobial therapy with trimethoprim-sulfamethoxazole (TMP-SMX) was promptly initiated. However, due to worsening thrombocytopenia, TMP-SMX was discontinued, and a combination of intravenous imipenem-cilastatin and oral minocycline was started. His immunosuppression was also reduced by discontinuatig mycophenolate. The patient’s course of illness was complicated by acute hemolytic anemia and pancytopenia with bone marrow biopsy showing changes of possible myelodysplastic syndrome. He also developed acute kidney injury which required admission to the intensive care unit. Due to the poor prognosis associated with his multiple medical problems, the patient elected to transition to hospice care, and he later expired.

## Discussion

*Nocardia* species are ubiquitous environmental microorganisms that are found worldwide and belong to a diverse group of bacteria known as aerobic actinomycetes [8]. They are obligate aerobic, partially acid-fast, catalase-positive, gram-positive bacilli with a branching filamentous form [9]. Nocardiosis has been reported to be increasing in the last two decades, potentially secondary to improvement in isolation of the organism and increased burden of immunocompromised patients [5]. The risk of developing nocardiosis after SOT varies with the type of organ transplanted and ranges from 1% to 4% after a heart or lung transplant to less than 1% after a kidney or liver transplant [10,11]. Although direct inoculation of the skin or soft tissues is one route of infection, the majority of nocardial infections are acquired via the respiratory tract [12]. Subsequent dissemination to other sites, such as the brain, skin, and subcutaneous tissues, is possible [12,13]. SOT recipients are at risk of disseminated *Nocardia* infections which can be an important cause of morbidity and mortality after transplantation. Protective immune responses to *Nocardia* infections are predominantly mediated by T-cells; thus, the impaired cell-mediated immunity associated with immunosuppressive therapy post-transplantation is a risk factor for acquiring *Nocardia* infections and for dissemination of the infection [8]. Other risk factors for acquiring *Nocardia* infections include glucocorticoid therapy, malignancy, human immunodeficiency virus infection, diabetes, and alcoholism [3]. In addition, nocardiosis can also occur in immunocompetent hosts, which accounts for approximately one-third of the infections [14]. Signs and symptoms of nocardiosis vary depending on the organs being affected. Symptoms often include fever, night sweats, unexplained weight loss, cough, and chest pain. When the central nervous system (CNS) is involved, patients may also experience headaches, confusion, and seizure [10]. Once disseminated, nocardiosis can resemble findings of metastatic malignancy [6,15]. One such report by Wuthrich et al. involved *N. paucivorans* infection in a renal transplant recipient, similar to our case [6]. The authors reported that a 59-year-old male with a history of renal transplant for kidney disease due to vasculitis presented with a painful thigh mass. He was subsequently found to have multiple pulmonary and chest wall lesions on imaging, and biopsy showed no evidence of malignancy, but culture grew *N. paucivorans *[6].

*N. paucivorans* is an uncommon, more recently described species of *Nocardia* that makes up a minority of isolates in *Nocardia* case series [5,16,17]. It may have a higher predilection for CNS dissemination [18] but limited data on clinical features and antimicrobial susceptibility testing are available. Sulfonamides, particularly TMP-SMX, have been the standard of therapy for nocardiosis for many years due to excellent activity against *Nocardia* in vitro and successful treatment outcomes [8]. However, it is important to accurately determine the species of *Nocardia *causing infection and obtain susceptibility testing whenever possible because there are differences in antimicrobial susceptibility patterns according to species [5,12]. Zhao et al. determined the susceptibility of 65 standard *Nocardia *isolates to 32 antimicrobial agents, but only one isolate was *N. paucivorans*. That isolate showed broad susceptibility to various standard anti-nocardial agents, including TMP-SMX, imipenem, ceftriaxone, linezolid, amikacin, fluoroquinolones, minocycline, and doxycycline. Schlaberg et al. found only 11 *N. paucivorans *isolates among 1,299 collected in a six-year period and found 100% susceptibility to TMP-SMX, amikacin, imipenem, ceftriaxone, and linezolid, but a 90% rate of non-susceptibility to amoxicillin-clavulanic acid [17]. TMP-SMX remains the treatment of choice for *N. paucivorans* infection as well as most other species [19].

For cerebral, disseminated, or severe pulmonary infection, the use of TMP-SMX in combination with other agents, such as amikacin or imipenem, as empiric therapy is recommended, and other agents such as minocycline, linezolid, ceftriaxone, or cefotaxime may be useful as alternative therapies [5,12,20]. Because TMP-SMX exacerbated our patient’s chronic thrombocytopenia, a combination of imipenem-cilastatin and minocycline was used as second-line therapy. Immunocompetent patients with pulmonary or non-CNS multifocal nocardiosis may require six months to one year of antimicrobial therapy to be adequately treated, while those with CNS involvement should receive at least one year of antimicrobial therapy together with appropriate clinical surveillance [5]. Patients who continue to be symptomatic despite antimicrobial therapy should be re-evaluated thoroughly for complications, such as primary drug resistance, inadequate serum antibiotic concentrations, poor penetration of the drug into the infected tissue compartment, or the presence of an abscess requiring surgical drainage [8]. High-risk individuals with lifelong immunosuppression including SOT recipients or hematopoietic cell transplant recipients should also be considered for secondary prophylaxis to prevent relapse or recurrence of nocardiosis [20]; however, breakthrough nocardial infections in patients on TMP-SMX prophylaxis have been described [5].

Establishing a diagnosis of nocardiosis in immunocompromised hosts is challenging for multiple reasons. As in this and other reports, infection by *Nocardia* spp. can mimic illnesses such as malignancy and tuberculosis [18]. Transplant recipients may be prescribed prophylactic antimicrobials, which can limit the sensitivity of clinical cultures, and they may have concomitant coagulopathy that makes sampling affected tissues prohibitively risky. In addition, the microbiologic culture of *Nocardia* can take weeks due to its fastidious nature and slower growth [21]. Moreover, as in the present case, cultures more likely to recover *Nocardia* (e.g., AFB or fungal cultures) may not be ordered on clinical specimens due to low suspicion of *Nocardia*. Delaying the diagnosis of nocardiosis has been associated with worse outcomes [8]. Therefore, there is an unmet need for novel, rapid, cost-effective, and noninvasive diagnostic methods for nocardiosis. mNGS of plasma cell-free DNA has emerged as an attractive diagnostic modality allowing broad-range pathogen detection, noninvasive sampling, and earlier diagnosis [7]. A recent report shows that mNGS is useful in the diagnosis of many viral, bacterial, fungal, and protozoal infections, including a case of *Nocardia cyriacigeorgica* pneumonia [22]. The current case also demonstrates the utility of mNGS in diagnosing disseminated nocardiosis, including identifying the species. This can assist in antimicrobial selection when the organism has not been cultured.

## Conclusions

SOT recipients are prone to unusual opportunistic infections. Disseminated nocardiosis can masquerade as a metastatic disease in SOT recipients. Indolent, unexplained constitutional symptoms, even in the absence of fever, should alert providers to the possibility of nocardiosis in SOT recipients. Nocardiosis due to *Nocardia paucivorans* is uncommon, and there are limited susceptibility data available. Prompt diagnosis and treatment of nocardiosis, including by mNGS, can result in successful patient outcomes and prevent unwanted consequences.
